# The Frequency of POCUS in the Treatment of Sepsis in the Emergency Department: A Retrospective Cohort Study

**DOI:** 10.24908/pocus.v8i1.15683

**Published:** 2023-04-26

**Authors:** M Bryan Dalla Betta, Dasia Esener, William Swanson, Andrew Kaddis, Felipe Aguayo Romero, J Matthew Fields

**Affiliations:** 1 Department of Emergency Medicine, Kaiser Permanente San Diego San Diego, CA USA; 2 Department of Emergency Medicine, Scripps Mercy Hospital San Diego, CA USA; 3 Department of Emergency Medicine, Baylor College of Medicine Houston, TX USA; 4 Department of Emergency Medicine, Thomas Jefferson University Philadelphia, PA USA

**Keywords:** POCUS, Sepsis, Ultrasound, Emergency Medicine, Point of care

## Abstract

**Background**: Point of care ultrasound (POCUS) is ubiquitous in the modern emergency department (ED). POCUS can be helpful in the management of patients with sepsis in many ways including determining the cause of sepsis, assessing fluid status, guiding resuscitation, and performing procedures. However, the frequency and manner in which POCUS is incorporated into the care of septic patients in community emergency medicine remains unclear. **Objective:** To evaluate POCUS frequency and exam types used in the care of patients with sepsis in two community EDs in Southern California. **Methods:** We performed a retrospective analysis of 5,264 ED visits with a diagnosis of sepsis at two community emergency departments between January 2014 and December 2018. Patients 18 years or older who were diagnosed with sepsis and had either lactate ≥ 4 mmol, a documented mean arterial pressure (MAP) < 65 mmHg, or a systolic blood pressure (SBP) < 90 mmHg were included. Charts were reviewed to determine if POCUS was used during the ED evaluation. Primary outcomes were frequency of POCUS use in the cohort, change in POCUS use over the study period, and the types of exams performed. **Results:** POCUS was used in 21% of encounters meeting inclusion criteria and was positively correlated with ED arrival year (OR = 1.09; CI 1.04, 1.15; p=0.001). The most common POCUS exam was ultrasound-guided central line placement, with the next most common exams being cardiac, followed by inferior vena cava (IVC). Only the frequency of cardiac, IVC, lung and Focused Assessment with Sonography in Trauma (FAST) exams were found to increase significantly over the study period. **Conclusions:** Total POCUS use increased significantly in this cohort of septic patients over the study period due to more cardiac, IVC, lung and FAST exams being performed.

## Background

Sepsis is a syndrome characterized by infection, widespread inflammation and organ dysfunction affecting millions of people in the United States and across the globe each year 1, 2, 3. Despite recent improvements in sepsis care, it is still associated with high rates of morbidity and mortality, accounting for nearly 270,000 deaths and treatment costs over $20 billion in the United States annually 2, 3. 

Although there is some disagreement regarding the optimal treatment of sepsis, current guidelines emphasize early diagnosis, antimicrobial administration, and intravenous fluid resuscitation for hypotension 2, as some studies have shown these interventions to improve outcomes 4, 5, 6. This emphasis on timely diagnosis and treatment, coupled with the many patients who initially present to the emergency department (ED) for their sepsis care, has made ED sepsis treatment the focus of multiple landmark sepsis studies 7, 8, 9. 

Paralleling advances in sepsis care, point of care ultrasound (POCUS) has become ubiquitous in the ED and across other specialties. In addition to procedural applications, POCUS can be used to evaluate patient hemodynamics and fluid status 10, 11, 12, 13, 14, assess cardiac function 15, 16, differentiate dyspnea 17, 18, 19, 20, 21, 22, and evaluate potential sources of infection 19, 23, 24, 25, 26, 27, 28.

Given the availability of POCUS and its ability to rapidly evaluate infection, cardiac function and fluid status, a role for it in the care of septic patients has been previously proposed 11, 23, 29. However, to our knowledge there is no widely accepted POCUS protocol specific to the septic patient, and the degree to which POCUS use has been integrated into sepsis care in community EDs is unknown. Our study examined five years of data to characterize the use of POCUS in patients with sepsis.

## Methods

We conducted a retrospective cohort study of patients with international classification of diseases (ICD)-9 and ICD-10 codes for sepsis and septicemia who presented to two community emergency departments in the San Diego metropolitan area from January 1, 2014 to December 31, 2018. Both departments are part of the same health network (Kaiser Permanente) and staffed by the same cohort of emergency physicians, which includes a three-year emergency medicine (EM) residency with 6 residents per year (established in 2014) and EM-based POCUS fellowship with 1-2 fellows per year (established 2015). The sites are self-described as “community” EDs meaning there is either no academic institution affiliation or patient care is largely provided by non-trainees. Despite having a residency and fellowship start during the study period, the vast majority of patients were cared for by non-trainees given the small number of trainees during that period. One of the two departments opened during the study period in 2016. The two departments are both located within 5 miles of each other in suburban portions of San Diego and have a similar patient population with a combined annual census of approximately 130,000 visits. The study was reviewed and approved by the Kaiser Permanente institutional review board and was granted a waiver of informed consent. 

Patients met criteria for inclusion if they were age 18 years or older, had an ED diagnosis of sepsis, and did not self-discharge against medical advice. In defining sepsis in our cohort, we adopted a modified definition of “severe sepsis”, which included at least one of the following additional inclusion criteria: any lactate ≥ 4 mmol/dL, any systolic blood pressure (SBP) < 90 mmHg, or any mean arterial pressure (MAP) < 65 mmHg. We chose this definition as opposed to the more recent Sepsis 3 guidelines, because our study evaluated the period from 2014 to 2018 when the former definitions were more commonly utilized in clinical practice 1. A higher lactate threshold of ≥ 4 mmol/dL was chosen to limit inclusion of patients with small lactate elevations who may not have been septic.

Charts for eligible encounters were abstracted to determine which patients received POCUS as part of their ED evaluation. Patients receiving POCUS were identified by electronic text search of the ED documentation within the electronic health record for the phrases “ultrasound”, “US”, “bedside US”, and “POCUS”; and by searching the emergency department’s cloud-based POCUS image archive (Telexy QPath) for studies with a matching medical record number and encounter date. Encounters positive for any search criteria were reviewed further by one of five study investigators using a standardized abstraction algorithm to confirm they received POCUS in the ED. If POCUS use was confirmed by investigator review, the exam type(s) were recorded based on ED documentation or archived exam images. If no exam type could be ascertained but POCUS use was clearly documented, the patient was deemed to have received POCUS for study purposes, and type was recorded as “unknown”. Encounters without evidence of POCUS by text search and without an image archive match were assumed not to have received POCUS during their ED evaluation and were not further reviewed. If text or image archive searches for an encounter were positive, but POCUS use could not be confirmed by chart review, the patient was deemed not to have received POCUS for study purposes.

Scanning protocol for all exam types was consistent with the American College of Emergency Physicians Emergency Ultrasound Imaging Criteria Compendium 30. A few notations to scanning protocols in the study institutions are as follows. The Focused Assessment with Sonography in Trauma (FAST) exam is frequently applied in non-trauma patients to evaluate for medical causes of free fluid in the peritoneum, pleural effusion, or pericardial effusion. Lung ultrasound protocol is to scan the anterior and axillary lung fields to evaluate for lung sliding and presence of B-lines. Our study evaluation of lungs for pneumothorax was categorized as a lung or pulmonary ultrasound exam and not as an extended FAST. Evaluation for sonographic signs of pneumonia is not routinely done by the physician cohort. Cardiac ultrasound and inferior vena cava (IVC) ultrasound are considered separate modalities. Evaluation of the IVC is performed in the subxiphoid or axillary location in two planes. IVC measurements are done both qualitatively and quantitatively with IVC collapse measurements performed 1-3 cm below where the IVC traverses the diaphragm or at the inlet of the hepatic veins. Cardiac ultrasound is performed using a phased array probe and acquired using subxiphoid, parasternal, and apical approaches for evaluation of pericardial effusion, cardiac function, presence of right heart strain, and evaluation of aortic outflow tract diameter. 

To evaluate the accuracy of the chart review, one reviewer randomly selected 189 previously reviewed encounters for re-review. The same review methodology was used on re-review of these 189 charts to confirm initial conclusions regarding POCUS use and exam type(s). 

### Statistical Analysis

We collected patient demographics and characteristics to determine what factors were correlated with POCUS use. Some patients had multiple ED encounters in the study cohort, and generalized estimating equations (GEE) were used to account for potential intra-patient correlation and to estimate the odds ratio of having POCUS by increasing ED arrival year. To address potential confounding between year and POCUS use, we utilized multivariable GEE models adjusted for age, sex, race/ethnicity, do-not-resuscitate (DNR) status, Charlson comorbidity index, insurance type, obesity, lactate ≥ 4 mmol, MAP < 65 mmHg, and SBP < 90 mmHg. 

In our physician group, IVC and Cardiac ultrasound are used to guide fluid management, whereas other modalities are used diagnostically or procedurally. To better understand how ultrasound is utilized in our cohort, we categorized POCUS exams a priori as resuscitative (Cardiac and IVC), diagnostic (Lung, FAST, Biliary, Aorta, Renal, Soft Tissue/Musculoskeletal, Bowel, DVT, OB/GYN) or procedural, and repeated multivariable GEE analyses using each POCUS category as the outcome with the same covariables. We then separately analyzed the most frequently used POCUS exam types to determine if their usage changed over time.

The kappa statistic was calculated to compare the performance of chart abstraction for assessing POCUS use and exam types. We used two-sided p-values with a threshold of 0.05 to indicate statistically significant results in this exploratory study. SAS version 9.4 (Cary, NC) was used to perform all analyses.

## Results

**Figure 1:**
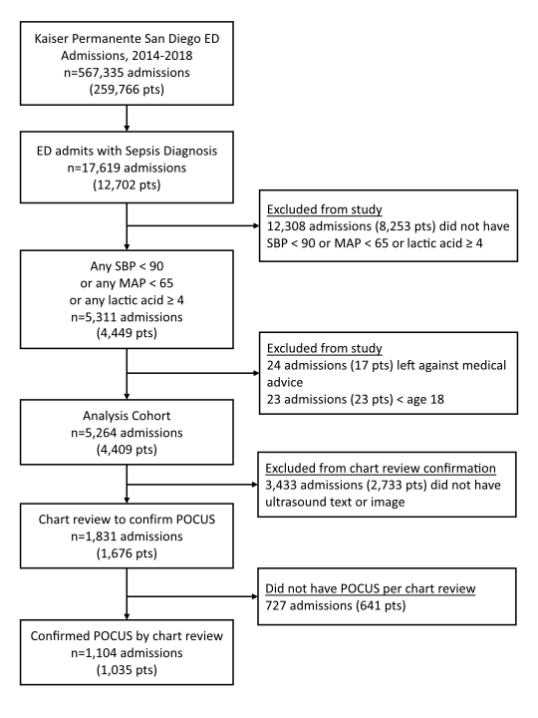
CONSORT diagram. Abbreviations: ED, emergency department; pts, patients; SBP, systolic blood pressure; MAP, mean arterial pressure; POCUS, point of care ultrasound.

There were 5,264 ED encounters for 4,409 patients that met inclusion criteria, with 13% (572) of patients contributing more than one encounter (Figure 1). Of the 5,624 encounters, 3,433 were excluded due to a lack of a POCUS image in the database and a negative result on text query of the ED documentation. The remaining 1,831 ED encounters underwent manual review of which 727 encounters were excluded due to lack of any documentation indicating a POCUS was performed. Approximately 21% (1,104) of encounters received POCUS as part of their ED evaluation and were included in the final analysis. The proportion of encounters getting POCUS during their ED care ranged from 18% in 2015 to 23% in 2018 (Table 1). Those receiving POCUS tended to be younger, with obese BMI, and had a non-DNR code status at time of ED arrival (Table 2).

**Table 1:** Odds ratios and frequency of select POCUS types by year.

	**Year of ED Arrival**						**Multivariable GEE**	
	**2014 (n=801)**	**2015 (n=978)**	**2016 (n=988)**	**2017 (n=1191)**	**2018 (n=1306)**	**Total** **(n= 5264)**	**Adjusted OR (95% CI)**	**p-value**
Any POCUS	151 (19%)	179 (18%)	221 (22%)	252 (21%)	301 (23%)	1104 (21%)	1.09 (1.04, 1.15)	**0.001**
Any procedural POCUS	107 (13%)	120 (12%)	140 (14%)	137 (12%)	153 (12%)	657 (13%)	0.97 (0.91, 1.03)	0.33
CVC POCUS	86 (11%)	104 (11%)	121 (12%)	111 (9%)	136 (10%)	558 (11%)	0.98 (0.92, 1.05)	0.58
Any resuscitative POCUS	23 (2.9%)	39 (4.0%)	68 (6.9%)	80 (6.7%)	113 (8.7%)	323 (6.1%)	1.31 (1.20, 1.42)	**<.0001**
Cardiac POCUS	17 (2.1%)	34 (3.5%)	61 (6.2%)	66 (5.5%)	98 (7.5%)	276 (5.2%)	1.32 (1.21, 1.44)	**<.0001**
IVC POCUS	12 (1.5%)	17 (1.7%)	39 (4.0%)	37 (3.1%)	66 (5.1%)	171 (3.3%)	1.35 (1.21, 1.52)	**<.0001**
Any diagnostic POCUS	30 (3.8%)	41 (4.2%)	53 (5.4%)	79 (6.6%)	93 (7.1%)	296 (5.6%)	1.21 (1.11, 1.32)	**<.0001**
Lung POCUS	4 (0.5%)	10 (1.0%)	27 (2.7%)	37 (3.1%)	51 (3.9%)	129 (2.5%)	1.52 (1.33, 1.74)	**<.0001**
FAST POCUS	10 (1.3%)	8 (0.8%)	13 (1.3%)	20 (1.7%)	25 (1.9%)	76 (1.4%)	1.19 (1.00, 1.41)	**0.048**
n: Total study encounters in each given year%: Percent of the total encounters in a given year who received each POCUS type (note: some encounters received multiple POCUS) p-value tests increasing or decreasing frequency from 2014-2018 Multivariable GEE is adjusted for age, sex, race/ethnicity, DNR before arrival, Charlson comorbidity index, insurance type, obesity, worst lactate, worst MAP, and worst SBP Abbreviations: CI, confidence interval; CVC, central venous catheter; FAST, Focused Assessment with Sonography in Trauma; GEE, generalized estimating equations; IVC, inferior vena cava; OR, odds ratio; POCUS, point of care ultrasound.								

**Table 2:** Patient Demographics and Characteristics.

	**POCUS** **(n=1104)**	**No POCUS (n=4160)**	**Total (n=5264)**
Age at ED arrival, mean (SD)	68.5 (15.9)	70.7 (16.9)	70.2 (16.7)
Female Sex	570 (52%)	2151 (52%)	2721 (52%)
Race/Ethnicity			
White	654 (59%)	2595 (62%)	3249 (62%)
Hispanic (all races)	268 (24%)	973 (23%)	1241 (24%)
Asian/Pacific Islander	114 (10%)	345 (8%)	459 (9%)
Black/African American	62 (6%)	235 (6%)	297 (6%)
Other	6 (<1%)	12 (<1%)	18 (<1%)
Spoken Language			
English	991 (90%)	3746 (90%)	4737 (90%)
Spanish	97 (9%)	337 (8%)	434 (8%)
Other	16 (1%)	77 (2%)	93 (2%)
Insurance type			
Medicare	694 (63%)	2820 (68%)	3514 (67%)
Medicaid	60 (5%)	205 (5%)	265 (5%)
Private	350 (32%)	1135 (27%)	1485 (28%)
Charlson comorbidity index, mean (SD)	5.0 (3.1)	4.8 (3.1)	4.9 (3.1)
BMI Category			
Normal (<25)	431 (39%)	1743 (42%)	2174 (41%)
Overweight (25 to <30)	288 (26%)	1171 (28%)	1459 (28%)
Obese (30+)	383 (35%)	1238 (30%)	1621 (31%)
Missing	2 (<1%)	8 (<1%)	10 (<1%)
DNR status before ED arrival	202 (18%)	999 (24%)	1201 (23%)
ED Arrival Year			
2014	151 (14%)	650 (16%)	801 (15%)
2015	179 (16%)	799 (19%)	978 (19%)
2016	221 (20%)	767 (18%)	988 (19%)
2017	252 (23%)	939 (23%)	1191 (23%)
2018	301 (27%)	1005 (24%)	1306 (25%)
Any Lactate ≥ 4*	389 (35%)	1061 (26%)	1450 (28%)
Any MAP < 65*	955 (87%)	3258 (78%)	4213 (80%)
Any SBP < 90*	865 (78%)	1956 (47%)	2821 (54%)
How Many Inclusion Criteria (Lactate, MAP, SBP) Met?			
Only 1 criteria	253 (5%)	2314 (56%)	2567 (49%)
2 criteria	597 (54%)	1577 (38%)	2174 (41%)
All 3 criteria	254 (23%)	269 (6%)	523 (10%)
*Encounters needed to meet at least 1 of these inclusion criteria for the study;percentages may not add up to 100% due to rounding. Abbreviations: BMI, body mass index; DNR, do not resuscitate; ED, emergency department; POCUS, point of care ultrasound; MAP, mean arterial blood pressure; SBP, systolic blood pressure; SD, standard deviation.			

In multivariate analyses (Table 3), increasing ED arrival year was associated with increased POCUS use (OR = 1.09, p = 0.001). Furthermore, obesity (OR = 1.28, p = 0.002), a higher Charlson comorbidity index (OR = 1.03, p = 0.03), lactate ≥ 4 (OR = 2.75, p < 0.0001), MAP < 65 (OR = 1.84, p < 0.0001), and SBP < 90 (OR = 4.24, p < 0.0001) were also found to be independently associated with increased POCUS use during ED stay. Conversely, a code status of DNR prior to ED arrival conferred lower odds of POCUS (OR = 0.74, p = 0.002). Increasing age also appeared to have an inverse association with any POCUS use but did not meet statistical significance in multivariable testing (p = 0.07).

**Table 3:** Univariable and multivariable analysis of patient characteristics influencing POCUS use.

	**Univariable GEE**		**Multivariable GEE**	
**Variable**	**OR (95% CI)**	**p-value**	**OR (95% CI)**	**p-value**
ED Arrival Year	1.08 (1.02, 1.13)	**0.004**	1.09 (1.04, 1.15)	**0.001**
Age, continuous	0.99 (0.99, 1.00)	**<.0001**	0.99 (0.99, 1.00)	0.07
Female vs Male	0.99 (0.87, 1.13)	0.88	1.03 (0.89, 1.19)	0.67
Hispanic vs White	1.08 (0.92, 1.28)	0.34	0.98 (0.82, 1.17)	0.82
Black/Other vs White	1.10 (0.84, 1.45)	0.49	0.96 (0.71, 1.30)	0.79
Asian vs White	1.31 (1.04, 1.64)	**0.02**	1.23 (0.96, 1.57)	0.10
DNR prior to admission	0.71 (0.60, 0.84)	**<.0001**	0.74 (0.62, 0.90)	**0.002**
Charlson index, continuous	1.03 (1.00, 1.05)	**0.02**	1.03 (1.00, 1.05)	**0.03**
Medicaid vs Medicare	1.14 (0.83, 1.58)	0.42	0.91 (0.63, 1.31)	0.61
Private vs Medicare	1.25 (1.08, 1.45)	**0.003**	1.07 (0.88, 1.30)	0.50
Obese vs Non-Obese BMI	1.26 (1.09, 1.45)	**0.002**	1.28 (1.10, 1.49)	**0.002**
Worst lactate ≥ 4	1.60 (1.39, 1.84)	**<.0001**	2.75 (2.33, 3.24)	**<.0001**
Worst MAP < 65	1.78 (1.47, 2.16)	**<.0001**	1.84 (1.48, 2.29)	**<.0001**
Worst SBP < 90	4.08 (3.49, 4.76)	**<.0001**	4.24 (3.59, 5.00)	**<.0001**
Abbreviations: BMI, body mass index; CI, confidence interval; DNR, do not resuscitate; ED, emergency department; GEE, generalized estimating equations; MAP, mean arterial blood pressure; OR, odds ratio; POCUS, point of care ultrasound; SBP, systolic blood pressure.				

When looking at different POCUS types, the most common exam performed was ultrasound-guided central line placement (Table 4). There were 558 instances of this exam, accounting for 37% of all POCUS performed during the study period. There was no increase in use of this exam over the study period (OR = 0.98, p = 0.58), nor was there any change in the amount of procedural POCUS performed overall (OR = 0.97, p = 0.33) (Table 1).

**Table 4:** POCUS exam types by year.

	**Year of ED Arrival**					
**POCUS Exam Type**	**2014 (n=174)**	**2015 (n=224)**	**2016 (n=327)**	**2017 (n=337)**	**2018 (n=438)**	**Total** **(n= 1500)**
**Total procedural POCUS**	**107 (61%)**	**121 (54%)**	**144 (44%)**	**138 (41%)**	**154 (35%)**	**664 (44%)**
CVC	86 (49%)	104 (46%)	121 (37%)	111 (33%)	136 (31%)	558 (37%)
IV_RN	12 (7%)	5 (2%)	1 (0%)	10 (3%)	9 (2%)	37 (2%)
IV_MD	1 (1%)	2 (1%)	8 (2%)	5 (1%)	3 (1%)	19 (1%)
Other Procedural	8 (5%)	10 (4%)	14 (4%)	12 (4%)	6 (1%)	50 (3%)
**Total resuscitative POCUS**	**29 (17%)**	**51 (23%)**	**100 (31%)**	**103 (31%)**	**164 (37%)**	**447 (30%)**
Cardiac	17 (10%)	34 (15%)	61 (19%)	66 (20%)	98 (22%)	276 (18%)
IVC	12 (7%)	17 (8%)	39 (12%)	37 (11%)	66 (15%)	171 (11%)
**Total diagnostic POCUS**	**37 (21%)**	**48 (21%)**	**82 (25%)**	**95 (28%)**	**114 (26%)**	**376 (25%)**
Lung	4 (2%)	10 (4%)	27 (8%)	37 (11%)	51 (12%)	129 (9%)
FAST	10 (6%)	8 (4%)	13 (4%)	20 (6%)	25 (6%)	76 (5%)
Biliary	10 (6%)	8 (4%)	12 (4%)	13 (4%)	9 (2%)	52 (3%)
Aorta	2 (1%)	7 (3%)	10 (3%)	4 (1%)	10 (2%)	33 (2%)
Renal	4 (2%)	6 (3%)	10 (3%)	2 (1%)	7 (2%)	29 (2%)
Soft tissue/Musculoskeletal	4 (2%)	7 (3%)	5 (2%)	5 (1%)	6 (1%)	27 (2%)
Bowel Appy	1 (1%)	1 (0%)	2 (1%)	1 (0%)	2 (0%)	7 (0%)
DVT	1 (1%)	0 (0%)	1 (0%)	1 (0%)	0 (0%)	3 (0%)
OBGYN	1 (1%)	0 (0%)	0 (0%)	1 (0%)	0 (0%)	2 (0%)
Other Diagnostic	0 (0%)	1 (0%)	2 (1%)	11 (3%)	4 (1%)	18 (1%)
**Unknown POCUS type**	**1 (1%)**	**4 (2%)**	**1 (0%)**	**1 (0%)**	**6 (1%)**	**13 (1%)**
n: number of POCUS exams performed that year %: percent of all POCUS exams performed that year Abbreviations: CVC, central venous catheter; DVT, Deep venous thrombosis; ED, emergency department; FAST, Focused Assessment with Sonography in Trauma; IV_RN, ultrasound guided IV placed by nurse; IV_MD, ultrasound guided IV placed by physician; IVC, inferior vena cava; OBGYN, Obstetrics and gynaecology; POCUS, point of care ultrasound;						

The next most common exams were cardiac (18%) and IVC (11%), both of which were categorized as resuscitative POCUS (Table 4). There was a statistically significant increase in use of these two exams over the study period (cardiac OR = 1.32, p < 0.0001; IVC OR = 1.35, p < 0.0001), as well as any resuscitative POCUS (OR = 1.31, p < 0.0001). 

Use of diagnostic POCUS also increased significantly (OR = 1.21, p < 0.0001), as did both lung (OR = 1.52, p < 0.0001) and FAST exams (OR = 1.19, p = 0.048), which were the most common diagnostic exams.

All other exam types accounted for small percentages of total POCUS exams and therefore were not evaluated in multivariable models due to their infrequent usage. The least common exams were DVT and obstetric/gynecologic exams. Thirteen exams (1%) were of undetermined type. To confirm the chart review findings, 189 charts were re-reviewed and found to have very high agreement for determining whether POCUS was performed (kappa = 0.97) and the specific POCUS exam types (kappa = 0.90).

## Discussion

To our knowledge, there is no consensus on how POCUS should be incorporated into sepsis care. Some recent studies on POCUS for the treatment of shock in the ED^31^ and for the treatment of sepsis in the ICU^29^ have shown no benefit. However, studies showing that POCUS leads to rapid diagnosis of sources of sepsis 23, increases physician certainty in sepsis treatment 32, 33, and reliably provides useful hemodynamic information 10, 11, 12, all suggest a role for POCUS in sepsis care. 

In our study, procedural POCUS exams were the most commonly performed exam type, with POCUS for central line placement being the single most frequent exam performed. This was an expected result as ultrasound guidance for central venous catheter (CVC) placement is routine in our ED, and a CDC recommendation 34. The use of POCUS for CVC placement did not change over the study period. It is also worthy to note that during the study period, an existing nurse-performed ultrasound-guided IV program was expanded, such that physicians were rarely called for IV access issues. Despite this, the number of ultrasound-guided CVCs in the cohort did not decrease, suggesting central access was required for reasons other than inadequate peripheral IV access. 

Data analysis also revealed increasing numbers of cardiac, IVC, lung and FAST exams being performed. These were the second through fifth most common POCUS exams respectively, and were the only exams with statistically significant OR after analysis (Table 1). We suspect several factors contributed to these findings. 

First, there was likely increasing familiarity with POCUS overall due to optional annual ultrasound training courses for the physicians, and the establishment of an emergency medicine residency and ultrasound fellowship at the study institution during this time frame. In one survey of 9 academic medical centers, it was found that establishment of an ultrasound fellowship program resulted in high utilization of ultrasound by residents 35. Increased bedside ultrasound availability may also have been a factor, as several new ultrasound machines were purchased for the emergency department during the study period. 

While these factors could explain an increase in all types of POCUS, only four exams were used more frequently. Determining why only these specific exams increased is beyond the scope of this study, but we hypothesize physicians may have used POCUS as a tool to guide resuscitation in these septic patients. We suspect this because the principal information provided by cardiac and IVC ultrasounds are estimations of cardiac function, presence of pericardial effusion, and IVC variability, which can relate information on a patient’s intravascular volume status. Given the low incidence of clinically significant pericardial effusion, we can infer that estimates of cardiac function and IVC variability were clinically useful to physicians treating this cohort of septic patients who had high rates of hypotension and IV fluid administration. 

The etiology of the increases in lung and FAST exams is less clear but may have been related to the diagnostic information they can provide in undifferentiated dyspneic and hypotensive patients, respectively. Both lung and FAST exams help to identify third spacing and free fluid in the lungs and abdomen. Additionally, these modalities may contribute to identifying the cause of sepsis such as pneumonia or infected ascitic fluid.

Characteristics of patients who did, and did not, receive POCUS as part of their sepsis care were also analyzed (Table 2). The two groups were found to be similar overall, but there were several important differences. We noted patients with an obese BMI or a higher Charlson comorbidity score to have higher odds of receiving POCUS, as did patients meeting an increasing number of study inclusion criteria (Table 3). This suggests that POCUS, like many other diagnostic resources, is more readily applied to patients with more severe illness. In these patients, decisions regarding fluid resuscitation, central line placement, or intubation are often made at the bedside with limited information. It is in this population that ultrasound can change decision making, as it often provides the only additional information after physical exam and point of care testing 14, 32, 34. 

It is unclear if obese patients received a particular type of POCUS more frequently than others, and we can only speculate as to why these patients were more likely to receive POCUS than those with a non-obese BMI. One explanation is decreased success of landmark guided peripheral IV placement in obese patients leading to increased use of POCUS-guided vascular access. Body habitus related limitations of the physical exam could have also contributed to their receiving more ultrasounds, as POCUS may have been used to further evaluate specific organ systems of interest. 

Similarly, patients with higher Charlson comorbidity index scores, by definition, had an increased chronic disease burden. This may have made them more susceptible to severe infection and thus more likely to need IV fluid therapy, hemodynamic assessment, and vasopressor support. In most instances, vasopressors require CVC placement which is typically done under ultrasound guidance. Similarly, we suspect a greater need for vasopressors and POCUS-guided CVC placement also explains the positive association between POCUS and patients meeting a greater number of study inclusion criteria. It is unclear if patients meeting more inclusion criteria or with higher Charlson scores received a particular type of POCUS exam more frequently.

Lastly, a code status of DNR prior to ED presentation was associated with decreased odds of receiving POCUS. We suspect these patients were more likely to receive selective care, and may have been less likely to accept certain therapies and invasive procedures, such as central line placement or other POCUS examination.

## Limitations

Our study has the same limitations as other retrospective studies, including limitations in data collection and abstractor bias. A standardized abstraction algorithm was used to decrease misclassification and abstractor bias, however since abstractors were not blinded, the latter remains a limitation. We attempted to identify every patient who received POCUS in the cohort by searching both the ED documentation and the emergency department’s POCUS image archive for evidence POCUS was used in a patient’s ED evaluation. Despite this, there may have been patients who received undocumented POCUS as part of their treatment, and as a result could not be identified. If this occurred in our cohort, it would result in an underestimation of POCUS use in these patients.

Beyond the study design, another source of confounding was the creation of an emergency medicine residency at the study site(s) in 2014. Because POCUS is part of the core competencies in emergency medicine training, the creation and growth of the residency may explain some increase in POCUS use.

Exclusion criteria requiring a diagnosis of sepsis and evidence of hypoperfusion decreased the study sample size. Given the poor specificity of systemic inflammatory response syndrome (SIRS) criteria, these inclusion thresholds were adopted to select for patients with a high likelihood of being septic, but the lactate, MAP, and systolic blood pressure criteria may have selected for patients closer to meeting current for septic shock rather than sepsis alone based on current guidelines. These inclusion criteria likely succeeded in decreasing the number of non-septic patients included in the study, however it is still possible patients with an underlying pathology other than sepsis may have been included. Our study design is concordant with the screening criteria for sepsis during the study period, and therefore the findings in this population should be comparable to other studies with similar definitions for sepsis. Additionally, the study was conducted prior to the COVID pandemic. Whether COVID has resulted in the utilization of POCUS is unclear and requires future investigation.

## Conclusions

Despite these limitations, we conclude that in this community ED setting, POCUS was increasingly used in the treatment of septic patients during the study period. This increase was primarily due to more cardiac and IVC exams being performed, and to a lesser extent, more lung and FAST exams being performed. We cautiously interpret this as evidence of increasing integration of POCUS into sepsis care in the community ED. Further studies are needed to test this hypothesis, determine if POCUS use affects clinical outcomes in patients with sepsis, and clarify the optimal role of POCUS in sepsis care. 

## Conflicts of Interest

None.
